# A comparative analysis of DNA barcode microarray feature size

**DOI:** 10.1186/1471-2164-10-471

**Published:** 2009-10-13

**Authors:** Ron Ammar, Andrew M Smith, Lawrence E Heisler, Guri Giaever, Corey Nislow

**Affiliations:** 1 Department of Molecular Genetics, University of Toronto, 1 King's College Circle, Toronto, Ontario M5S 1A8, Canada; 2 Banting and Best Department of Medical Research, University of Toronto, 112 College Street, Toronto, Ontario M5G 1L6, Canada; 3 Terrence Donnelly Centre for Cellular and Biomolecular Research, University of Toronto, 160 College Street, Toronto, Ontario M5S 3E1, Canada; 4 Department of Pharmaceutical Sciences, University of Toronto, 144 College Street, Toronto, Ontario M5S 3M2, Canada

## Abstract

**Background:**

Microarrays are an invaluable tool in many modern genomic studies. It is generally perceived that decreasing the size of microarray features leads to arrays with higher resolution (due to greater feature density), but this increase in resolution can compromise sensitivity.

**Results:**

We demonstrate that barcode microarrays with smaller features are equally capable of detecting variation in DNA barcode intensity when compared to larger feature sizes within a specific microarray platform. The barcodes used in this study are the well-characterized set derived from the Yeast KnockOut (YKO) collection used for screens of pooled yeast (*Saccharomyces cerevisiae*) deletion mutants. We treated these pools with the glycosylation inhibitor tunicamycin as a test compound. Three generations of barcode microarrays at 30, 8 and 5 μm features sizes independently identified the primary target of tunicamycin to be *ALG7*.

**Conclusion:**

We show that the data obtained with 5 μm feature size is of comparable quality to the 30 μm size and propose that further shrinking of features could yield barcode microarrays with equal or greater resolving power and, more importantly, higher density.

## Background

Genome-wide studies often measure changes in the abundance of all gene products over a period of time or under varying conditions. Microarrays have made these studies possible by enabling researchers to monitor all known genes of an organism simultaneously to detect patterns of gene activity [1], alternative splicing variants [2] the presence of single nucleotide polymorphisms [3], the presence of copy number variants and [4] DNA binding sites of diverse proteins [5], among others. One application of microarrays that our laboratory has focused on is the parallel identification of individual molecular barcoded gene deletion mutants grown competitively in pools [6,7]. Through the efforts of the Yeast Deletion Consortium, a Yeast KnockOut (YKO) collection was constructed consisting of approximately 6,000 heterozygous gene deletions (>96% of all annotated open reading frames), of which over 1,100 are known to be essential for growth [7]. The remaining ~5,000 genes are nonessential, created as homozygous deletions and *MAT*αand MATα deletion collections. These collections were made by systematic replacement of each gene from start to stop codon by mitotic recombination with a molecular barcoded resistance cassette. Each cassette contains both an upstream barcode (uptag) and a downstream barcode (downtag) that differ in their 20-mer sequence [7]. Drug sensitivity assays, combined with DNA barcode microarrays, were able to reveal genomic profiles for both the drug's targets through **H**aplo **I**nsufficiency **P**rofiling (**HIP**) and pathways that buffer the drug target pathway through **HO**mozygous deletion **P**rofiling (**HOP**) [8,9].

Microarrays are made up of thousands to millions of microscopic "features", clusters of identical oligonucleotide probes, which are used to detect hybridized gene products. The microarrays used for HIPHOP assays have gone through several iterations of development, beginning with a feature size of 103 μm on the TAG1 array which consisted of 20 bp (base pair) probes [6,8]. The *S. cerevisiae *cassette was originally designed for detection using the TAG1 microarray, which used 20 bp-long oligonucleotide probes. Current Affymetrix microarrays use up to 25 bp probes to detect complementary DNA sequences, and this length is more appropriate for newer barcoded collections as it improves hybridization specificity and increases the number of resolvable potential barcodes [10]. The features on these chips were subsequently miniaturized to 30 μm and provided full deletion pool coverage on the TAG3 array (P/N 510318) [7]. The current TAG4 chips (P/N 511331) with 8 μm feature sizes were designed for improved performance and affordability. This scheme omitted uninformative probes present on previous tag arrays and added five replicates to report non-uniform hybridization and allow adjustment of intensities accordingly [11]. No smaller yeast deletion pool barcode microarray exists due to manufacturing size constraints, however, these barcode probes are also present on the 5 μm yeast whole genome tiling array (S288c genome tiling microarray; P/N 520055) representing 0.25% of the total 6.5 million probes on this array [12]. The area of the features scale quadratically, such that the tiling array features at 5 μm on a side correspond to 25 μm^2^, and TAG3 features at 30 μm on a side correspond to 900 μm^2^, or 36 times the area of the tiling features. It is important to note that all arrays have the same oligonucleotide probe density of approximately 4,000 probes/μm^2 ^(personal communication with Affymetrix technical support).

## Methods

Yeast deletion pools were thawed from frozen stocks and heterozygote essential gene deletion mutants were grown for 20 generations, while homozygous deletion mutants were grown for 5 generations as described [13]. After growth, heterozygous essential deletion mutants were mixed with correspondingly treated homozygous non-essential deletion mutants. Genomic DNA was isolated and molecular barcodes amplified by PCR. Amplicons were then hybridized to microarrays over night, washed, stained and scanned the following day. For further details regarding sample preparation and data analysis, consult Pierce *et al *[14] and Hoon *et al *[13].

We performed a HIPHOP screen (pooled heterozygous essential strains and homozygous deletion non-essential strains) with tunicamycin treatment (IC_10-20 _= 0.35 μM). Tunicamycin is a known glycosylation inhibitor, targeting the yeast essential gene *ALG7 *[15-17], which encodes UDP-N-acetyl-glucosamine-1-P transferase, a vital protein in the dolichol pathway of protein asparagine-linked glycosylation [18,19]. Upon treatment with tunicamycin, unfolded proteins remain in the ER (endoplasmic reticulum) [20]. A sample treated with 2% DMSO was used as a control. Yeast pools were grown in liquid culture in 48 well plates in a shaking spectrophotometer interfaced to liquid handling robots. After the cells had grown for the desired number of generations, corresponding to a specific optical density (OD), they were robotically harvested [14]. Genomic DNA was isolated from each pool, and the DNA barcodes were amplified by PCR using common primers. These barcodes were subsequently hybridized to three generations of barcode microarrays: the aforementioned TAG3, TAG4 and *S. cerevisiae *whole genome tiling arrays. Each chip was prepared using the optimal hybridization and wash/stain protocols recommended for that array type. Deletion strain abundance was resolved by averaging scanned downtag and uptag intensities for each strain and comparing intensities between the tunicamycin-treated pool and the DMSO-treated pool [14] (see Additional File 1).

## Results and Discussion

**Figure 1:**
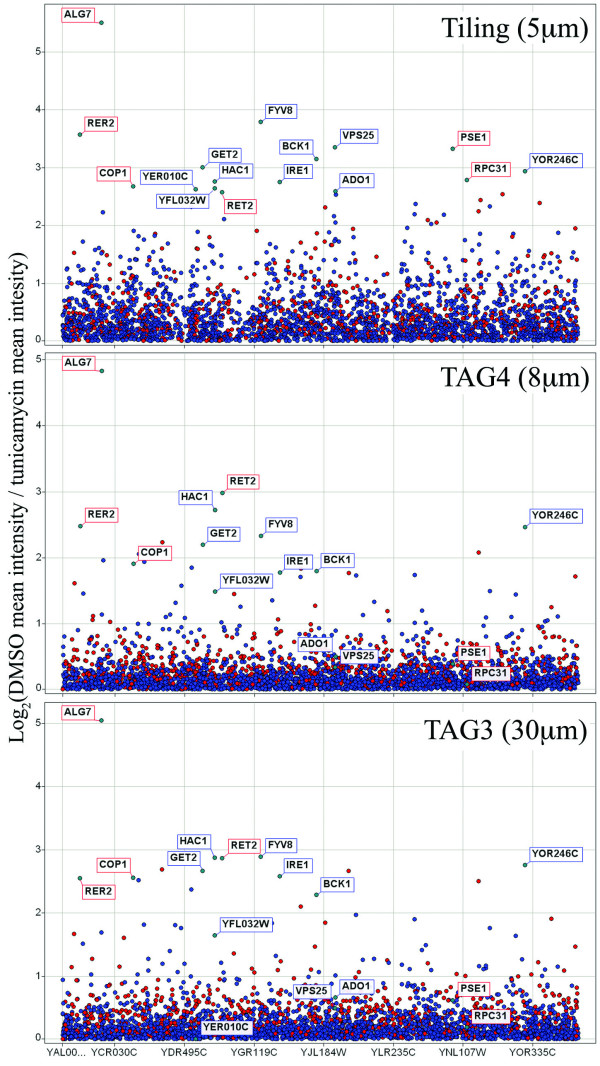
**Identifying tunicamycin targets on three microarray generations**. Barcode intensity data are normalized according to a DMSO reference treatment. Blue dots represent non-essential genes, red dots represent essential genes and grey dots are genes that are not annotated. Log_2 _ratios are calculated as a measure of change in barcode intensity (vertical axis) across all genes (horizontal axis). Ratios below 0 have been removed for clarity. Log_2 _scales differ based on optimal dynamic range between baseline and *ALG7*. Higher ratios correspond to greater abundance of barcode from reference to treatment. In all three analyses, *ALG7 *was correctly identified as the primary target of tunicamycin. Several additional genes previously determined to be resistant to tunicamycin, were most discernibly identified in the tiling data, but less so using TAG4 (the current microarray standard) and TAG3. These include *ADO1*, *BCK1*, *FYV8*, *GET2*, *HAC1 *and *IRE1*. Furthermore, the genes *COP1 *and *RER2*, known to be involved in ER to Golgi vesicle-mediated transport, showed up as sensitive to tunicamycin in our screen.

All three microarray generations, the TAG3, TAG4 and *S. cerevisiae *whole genome tiling arrays, identified *ALG7 *as the primary target of tunicamycin, as expected (Figure 1). The tiling array also identified several other genes as additional potential targets. This list of targets includes *ADO1*, *FYV8*, *GET2*, *HAC1 *and *IRE1*, all of which have been shown to be sensitive to tunicamycin when knocked out, as well as *BCK1*, a gene which has previously been shown to be resistant to tunicamycin when overexpressed [19,21-24]. In particular, *ADO1 *is a prime example of a gene deletion strain exhibiting increased sensitivity on the tiling array, since it is detected at a log_2 _ratio of 2.59 in the tiling array data, but at 0.50 and 0.66 in the TAG3 and TAG4 data, respectively. In addition to known sensitive strains, our screen identified *COP1 *and *RER2*, which are involved in ER to Golgi vesicle-mediated transport (see Table 1 for summary of sensitive strains) [25,26]. As with most sensitive strains, these genes were detected at slightly higher levels on the tiling array than on the other array generations. The tiling array appears to have slightly higher variance in its log_2 _ratios than the other arrays (standard deviation of 0.58 in tiling, compared to 0.37 and 0.43 in TAG4 and TAG3 arrays, respectively). We determined this to be due to its increased sensitivity to hybridized barcode abundance since sometimes strains that appear sensitive on the tiling array, fall into the background signal of the other arrays, as with *ADO1*. It is reassuring to observe both the primary target of tunicamycin and genes annotated as sensitive to tunicamycin in our results. Additionally, we also identified genes associated with the endoplasmic reticulum and involved in the unfolded protein response because tunicamycin promotes protein misfolding.

**Table 1:** Gene targets of tunicamycin identified in the tiling array experiment.

***ORF***	***Gene Name***	***GO Biological Process***	***tunicamycin treatment relevance***
YJR105W	*ADO1*	purine base metabolic process	knockout sensitive to tunicamycin [23]

YBR243C	*ALG7*	protein amino acid N-linked glycosylation *and others*	known target of tunicamycin [15-17]

YJL095W	*BCK1*	endoplasmic reticulum unfolded protein response *and others*	knockout sensitive [21-23], overexpressor resistant [21] to tunicamycin

YDL145C	*COP1*	ER to Golgi vesicle-mediated transport *and others*	involved in ER to Golgi vesicle-mediated transport [25]

YGR196C	*FYV8*	unknown	knockout sensitive to tunicamycin [21]

YER083C	*GET2*	protein insertion into ER membrane *and others*	knockout sensitive to tunicamycin [24]

YFL031W	*HAC1*	specific RNA polymerase II transcription factor activity *and others*	knockout sensitive to tunicamycin [21,23]

YHR079C	*IRE1*	endoplasmic reticulum unfolded protein response *and others*	knockout sensitive to tunicamycin [21,23]

YOR246C	*N/A*	unknown	unknown

YFL032W	*N/A*	unknown	likely deletes *HAC1 *promoter [19]

YER010C	*N/A*	unknown	interacts with kinases Ptk2, Tpk1 [30]

YMR308C	*PSE1*	protein import into nucleus *and others*	interacts with Ulp1, regulating ubiquitination [31]

YBR002C	*RER2*	ER to Golgi vesicle-mediated transport *and others*	involved in ER to Golgi vesicle-mediated transport [26]

YFR051C	*RET2*	ER to Golgi vesicle-mediated transport *and others*	interacts with Bre5, Hsc82, Hsp92 [31-33] which are involved in protein processing

YNL151C	*RPC31*	transcription from RNA polymerase III promoter	interacts with Mms1, Shp1, Ubi4, regulating ubiquitination [34,35]

YJR102C	*VPS25*	ubiquitin-dependent protein catabolic process via the multivesicular body sorting pathway *and others*	involved in ubiquitin-dependent protein catabolism [36]

Because the tiling array has millions of probes, only a few thousand of which are barcode probes, we hypothesized that non-specific hybridization of barcode DNA to the genome tiling probes could potentially contribute to noise in target identification. This may have been problematic because the tiling probes were not designed for explicit use with the barcode probes, which could lead to unanticipated cross-hybridization of barcode samples to tiling probe features. To determine if non-specific binding was a factor in our experiments, we co-hybridized barcode DNA with unlabeled digested genomic DNA (gDNA). The digested gDNA (20-150 bp) competitively hybridized to tiling probes of the array to which barcodes may have had a non-specific affinity. We asked if the addition of gDNA could result in an increase of specific binding of barcodes to barcode probes, yielding a HIPHOP profile with greater dynamic range and more distinct targets (making the millions of tiling probes unavailable for barcode hybridization) analogous to the addition of salmon or herring sperm to a Southern blot to prevent non-specific hybridization [27,28]. However, in practice, we found that the addition of gDNA did not improve resolution of the target *ALG7 *when compared to a microarray without competitive gDNA co-hybridization (Additional File 2).

**Figure 2:**
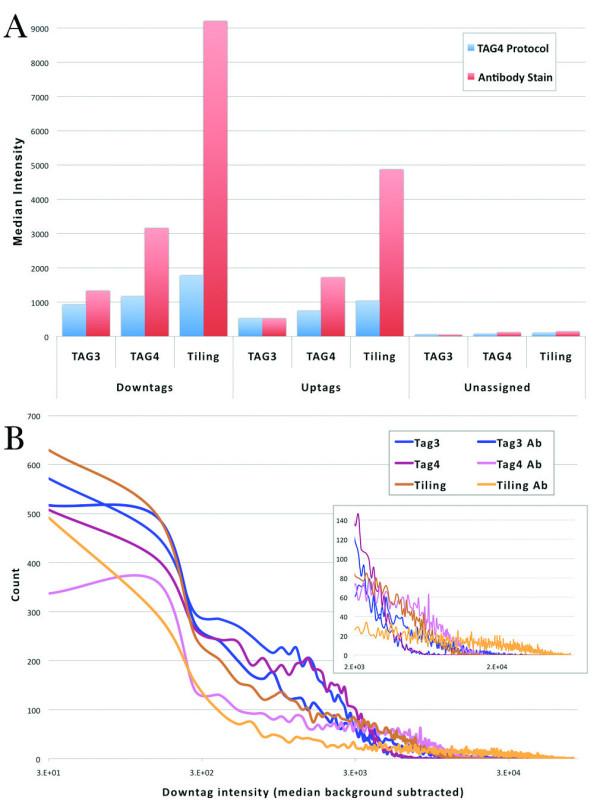
**A) Median intensity for all barcodes, including downtags, uptags and unassigned barcodes (used to measure background)**. Median is used due to non-normal intensity distributions (see B). Tiling intensities are consistently higher than TAG4, which are higher than TAG3. This trend is intensified by the addition of antibody staining. Downtags are consistently higher than uptags, as previously described [14]. Background intensity on all three generations is similar. B) Distributions of downtag intensity. Downtag intensity axis is shown on a logarithmic scale. Magnified view of high intensity values in inset. TAG3 and TAG4 arrays have more downtags at a lower intensity than the tiling array. As expected, after antibody staining, intensities were amplified, and the distributions have longer tails.

Our initial experiments used protocols for each microarray that were optimized for that particular technology. For example, each array type has particular hybridization, washing and staining protocols. To minimize the effect of these subtle variations and to accurately compare intensity data across array generations, we hybridized a reference sample (treated with 2% DMSO) to TAG3, TAG4 and tiling microarrays and applied TAG4 wash protocols to each array type. The hybridization conditions were fixed so that we could be certain that any changes we observed were attributed solely to feature size and not protocol variation. We scanned the microarrays following this protocol, and subsequently applied the tiling array antibody stain wash step to all three chips and, once again, scanned them. In this manner, each array was treated identically. In general, we observed median downtag intensity was higher than median uptag intensity (Figure 2), an observation that was also reported by Pierce *et al *[11,14]. In addition, the median intensities differed across generations, with TAG3 intensity lower than TAG4 intensity, which was lower than tiling intensity.

**Figure 3:**
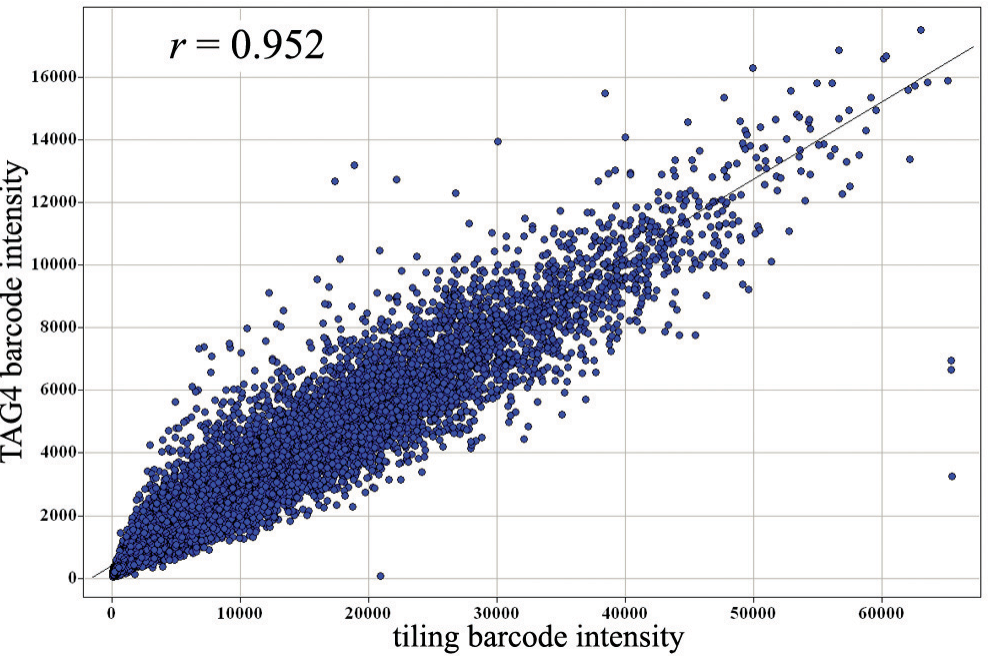
**TAG4 and tiling array data correlation after antibody staining**. This example shows that the signal intensity for common barcodes between TAG4 and tiling arrays are highly correlated (*r *= 0.952), demonstrating that tiling arrays are as accurate as TAG4 arrays when determining relative signal intensity (compared to a DMSO reference on the same chip generation).

We found that TAG4 and tiling array intensities were very highly correlated (Tables 2 and 3; example in Figure 3). This correlation increased slightly once the arrays had been antibody stained during the tiling wash protocol. In contrast, TAG3 intensities did not correlate as well with either TAG4 or tiling, and this decreased significantly after antibody staining. However, this low correlation is unlikely to affect identification of drug targets on TAG3 arrays, as these strains are often the most distinguishable from the background, as shown previously (Figure 1).

**Table 2:** Pearson correlation coefficients (*r*) across microarray generations without antibody (Ab) stain.

***-- Ab***	Tiling	TAG4	TAG3
TAG3	0.733	0.751	-

TAG4	0.927	-	
	
Tiling	-		

TAG4 and tiling data are highly correlated, and this increases with antibody staining (compare Table 3). The opposite trend is observed between TAG3 and TAG4 or tiling, where antibody staining exacerbates the effect. Correlation of barcode intensity is vital because when both treatment and reference results from a single generation are correlated across generations, target identification should be almost identical.

**Table 3:** Pearson correlation coefficients (*r*) across microarray generations with antibody (Ab) stain.

**+ *Ab***	Tiling	TAG4	TAG3
TAG3	0.605	0.642	-

TAG4	0.952	-	
	
Tiling	-		

TAG4 and tiling data are highly correlated, and this increases with antibody staining (compare Table 2). The opposite trend is observed between TAG3 and TAG4 or tiling, where antibody staining exacerbates the effect.

The relatively recent design of the TAG4 microarray includes five replicates of each barcode probe [11]. However, we noticed that intensity values do not vary greatly between these replicates, and, therefore, a minimum of three replicates should be included to allow for appropriate trim mean calculations and masking of unusable barcode probes [14]. This finding confirms an earlier assertion by Pierce *et al*. that suggests that the minimum number of replicates required to achieve high correlation is three replicates, and that the increase in correlation from the fourth and fifth replicates is marginal [11]. Although the TAG3 and tiling results contain only single data points for each barcode and are able to determine *ALG7 *as the primary target of tunicamycin (Figure 1), replicate data points are advised to accommodate hybridization, washing and staining inconsistencies.

## Conclusion

Here we present a systematic comparison of the behavior of 12,000 20 bp barcode probes at three feature sizes. Counter to our expectation, we found that the smallest features, representing less than 1/30 the space of the largest features, perform best in terms of signal intensity and in their ability to identify drug targets in complex pooled assays. We show that microarrays with reduced feature size are equally able to assess DNA barcode abundance when compared to barcode microarrays with larger features. An increased sensitivity was also observed with arrays with smaller features. They identified a previously described target of tunicamycin with greater confidence than the microarrays with greater feature size.

A widely held opinion is that next generation DNA sequencing technologies will replace microarrays in gene product detection [29]. However, microarrays can still increase genome coverage by decreasing feature sizes to as small as 1 μm because current microarray scanners can detect probe intensities at sub-μm resolution. In theory, such reductions in feature size could yield microarrays with approximately 202 million probes/chip (compared to 6.5 million using 5 μm features). Such probe densities would rival next generation sequencing technologies in terms of genome coverage.

## Competing interests

The authors declare that they have no competing interests.

## Authors' contributions

RA, AMS, GG, CN conceived of the project and designed experiments. RA and AMS performed the experiments. RA, AMS, LEH, GG, CN analyzed the data. RA, AMS, GG, CN wrote the paper.

## Supplementary information

Affymetrix microarray library files for the TAG3, TAG4 and tiling arrays are available at .

The supplementary figure displays the tiling array profiles when the DMSO and tunicamycin treatment chips are hybridized with the barcodes alone or with the addition of gDNA.

## Supplementary Material

Additional file 1 **supplementary_data**. A collection of processed microarray data corresponding to the experiments from the manuscript. Experimental details are included in the individual file names within the collection. Click here for file

Additional file 2 **Supplementary Figure**. A figure displaying the tiling array profiles when the DMSO and tunicamycin treatment chips are hybridized with the barcodes alone or with the addition of gDNA. Click here for file
