# Supplementary figures and images for: A comparative analysis of DNA barcode microarray feature size
Source: BMC Genomics. 2009 Oct 13;10:471. doi: 10.1186/1471-2164-10-471 (PMC2765990; doi:10.1186/1471-2164-10-471)

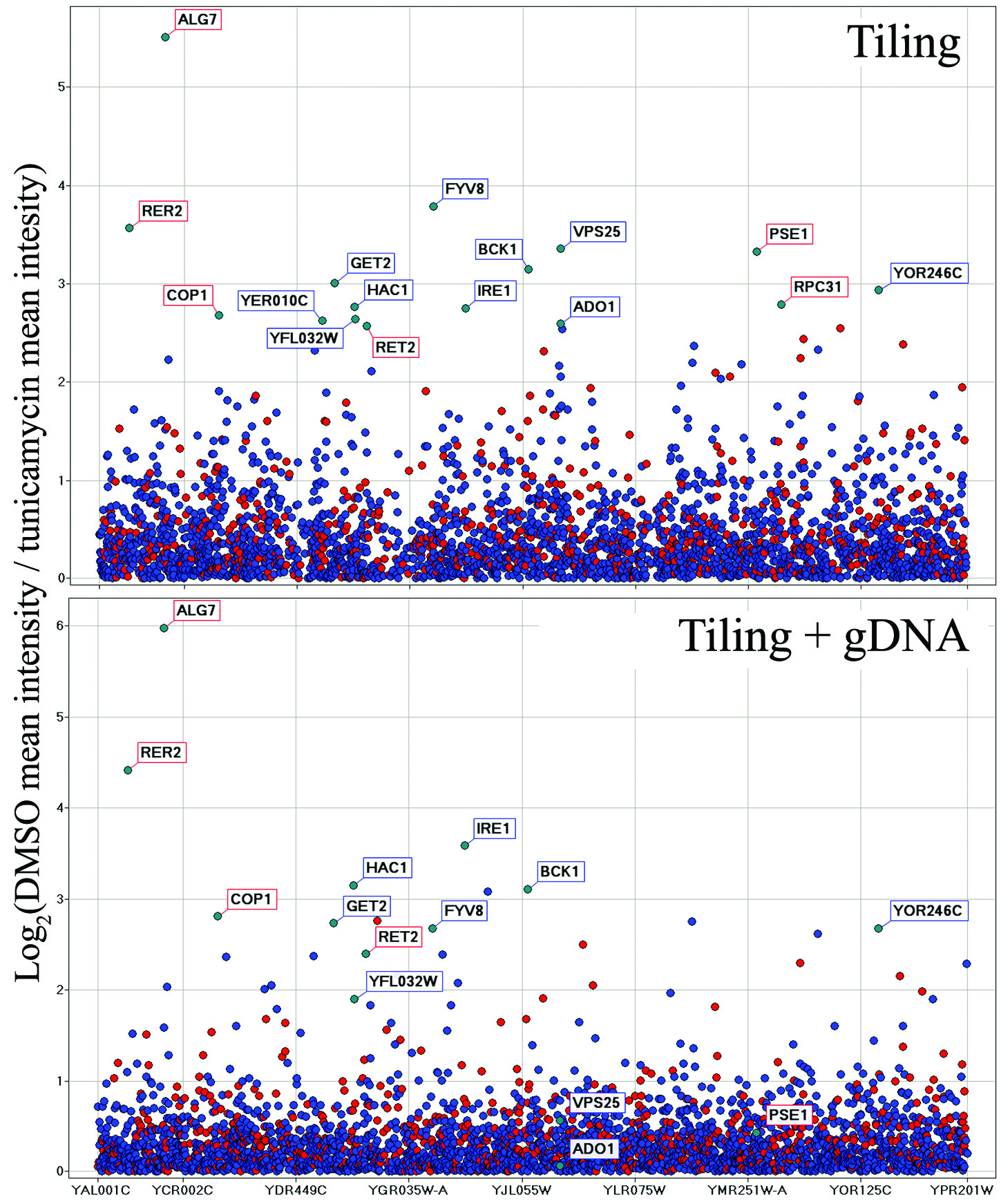

Supplement: Additional file 2 — Supplementary Figure. A figure displaying the tiling array profiles when the DMSO and tunicamycin treatment chips are hybridized with the barcodes alone or with the addition of gDNA. [file 1471-2164-10-471-S2.JPEG]
